# Genetically Encoded Fluorescent Biosensors Enable Noninvasive Real-Time Visualization of Nitrate Dynamics in Intact Living Plants

**DOI:** 10.3390/bios16050243

**Published:** 2026-04-26

**Authors:** Li Zhang, Qing Xu, Changxu Wang, Jinfeng Wang, Jing Yue, Yin Lu, Guangle Zhang, Lixue Yuan, Yonghua Wang, Bo Yu, Guozhang Kang

**Affiliations:** 1 The National Engineering Research Center for Wheat, Henan Agricultural University, Zhengzhou 450046, China; zhangli@stu.henau.edu.cn (L.Z.); xuqing@stu.henau.edu.cn (Q.X.); wangchangxu@stu.henau.edu.cn (C.W.); jinfengwang@stu.henau.edu.cn (J.W.); yuejing@stu.henau.edu.cn (J.Y.); luyin@stu.henau.edu.cn (Y.L.); zhangguangle@stu.henau.edu.cn (G.Z.); yuanlixue@stu.henau.edu.cn (L.Y.); wangyonghua@henau.edu.cn (Y.W.); 2 The State Key Laboratory of High-Efficiency Production of Wheat-Maize Double Cropping, Henan Agricultural University, Zhengzhou 450046, China; 3 Functional Crop Engineering Center in Henan Province, Henan Agricultural University, Zhengzhou 450046, China

**Keywords:** nitrate, biosensor, plant, noninvasiveness, visualization

## Abstract

Nitrate (NO_3_^−^) serves as a pivotal molecule with dual functions in nutrient supply and signaling during plant growth and development. Precise monitoring of its spatiotemporal dynamics in planta is therefore essential for dissecting the regulatory mechanisms underlying plant nitrogen metabolism. However, conventional nitrate detection methods suffer from inherent limitations, including destructive sampling, insufficient spatiotemporal resolution, and an inability to achieve real-time whole-plant monitoring. Here, we report a genetically encoded nitrate biosensor, designated NitNRCL1, constructed using a split firefly luciferase complementation system. Functional validation in both prokaryotic and eukaryotic systems demonstrates that NitNRCL1 responds to changes in nitrate availability and generates stable chemiluminescent signals in bacteria and diverse plant species. Importantly, NitNRCL1 enables non-invasive, real-time, and whole-plant monitoring of nitrate levels in living plants. Using NitNRCL1, we successfully imaged the spatiotemporal dynamics of nitrate signaling in *Arabidopsis thaliana*. Collectively, our findings establish NitNRCL1 as a robust and novel tool for investigating nitrate transport, signaling, and metabolic pathways in plants. This biosensor advances our mechanistic understanding of plant nitrate biology and provides a technical foundation for breeding nitrogen-use-efficient crops and developing precision fertilization strategies.

## 1. Introduction

Nitrate (NO_3_^−^) serves as the primary form of inorganic nitrogen absorbed by plants, exhibiting rapid root uptake and representing the preferred nitrogen source for the majority of crop species. As an indispensable macronutrient, NO_3_^−^ participates in the biosynthesis of critical biomolecules including chlorophyll, amino acids, proteins, and nucleic acids, thereby directly modulating photosynthetic efficiency and crop productivity [1]. Furthermore, optimal endogenous NO_3_^−^ concentrations facilitate the acquisition of other mineral nutrients such as phosphorus and potassium, ultimately enhancing plant nutrient use efficiency. Beyond its role as a fundamental nutritional substrate, NO_3_^−^ functions as a pivotal signaling molecule governing global plant growth and developmental programs, characterized by three hallmark properties: rapid transient responsiveness, metabolic independence, and genome-wide transcriptional reprogramming [2,3]. Distinct physiological responses are triggered in planta within seconds to 1 h following NO_3_^−^ application [4]. Recent advances employing biosensor technology and in vivo real-time imaging have revealed that calcium ions and cytokinins synergistically mediate long-distance nitrogen signaling between plant roots and shoots, enabling precise coordination of whole-plant nitrogen use efficiency and holistic growth trajectories [5,6]. As an irreplaceable metabolite with dual nutritional and signaling functionalities in plants [7], NO_3_^−^ executes unique and non-redundant physiological roles.

Plant NO_3_^−^ uptake constitutes a tightly regulated, spatiotemporally coordinated active metabolic process encompassing three sequential steps: uptake, translocation, and assimilation, which relies on an intact transmembrane transport cascade spanning from the rhizosphere soil to the interior of plant cells [3,8]. The concentration of NO_3_^−^ in soil solution fluctuates drastically across a broad range from micromolar to millimolar levels [9]. To adapt to this wide concentration spectrum, plants have evolved two functionally distinct NO_3_^−^ transport systems mediated by evolutionarily conserved transporter families: the NRT1/NPF family and the NRT2 family. Specifically, the high-affinity NO_3_^−^ transport system (HATS) displays a Michaelis constant (*K*) of approximately 50 μM, whereas the low-affinity NO_3_^−^ transport system (LATS) exhibits a *K* of roughly 5–12 mM [10,11]. These two systems collectively enable plants to efficiently acquire NO_3_^−^ across the entire physiologically relevant concentration range. Following cellular uptake, NO_3_^−^ undergoes long-distance root-shoot translocation and subsequent reallocation via the cyclic vascular network, with upward transport through the xylem and downward redistribution through the phloem [3,12,13]. Subcellular partitioning of NO_3_^−^ directly dictates its assimilation efficiency: the vacuole acts as the primary NO_3_^−^ storage compartment, accounting for 60–80% of total cellular NO_3_^−^ content [14], while the cytoplasm serves as the exclusive site for NO_3_^−^ metabolism [15]. Dynamic homeostasis between these two compartments is maintained by tonoplast-localized NO_3_^−^ transporters, which stabilize cytoplasmic NO_3_^−^ concentrations at 1–5 mM [15,16] to precisely match the catalytic activity requirements of nitrate reductase. Despite substantial progress in dissecting the core molecular mechanisms and regulatory networks underlying plant NO_3_^−^ uptake, the spatiotemporal dynamic crosstalk between NO_3_^−^ uptake and utilization, diverse environmental signals, and downstream chemical signaling cascades remains poorly understood.

Conventional NO_3_^−^ detection techniques, including colorimetry [17], ion chromatography (IC) [18], radioisotope tracing [19], and high-performance liquid chromatography-tandem mass spectrometry (HPLC-MS) [20], enable accurate quantitative data but are destructive to plant specimens and lack the spatiotemporal resolution required to monitor dynamic NO_3_^−^ fluctuations in living plants. In vivo detection approaches such as ion-selective microelectrode (ISME) analysis offer micrometer-scale high spatial resolution [21], yet fail to effectively capture the spatiotemporal dynamics of NO_3_^−^ levels in biological samples.

Fluorescence resonance energy transfer (FRET)-based biosensors enable optical probing of biological tissues with high spatiotemporal resolution, leveraging their exceptional subcellular resolution and millisecond-scale response kinetics [22]. However, this technology is intrinsically reliant on microscopic imaging and high-intensity excitation illumination, rendering long-term in vivo monitoring and whole-plant detection of plant samples challenging. To circumvent this limitation and satisfy the pressing need for real-time, whole-plant, high-resolution monitoring of NO_3_^−^ dynamics in planta, we developed a genetically encoded NO_3_^−^ biosensor, designated NitNRCL1, which employs split firefly luciferase (Fluc) as a signal transducer [23]. Designed for non-invasive, longitudinal imaging at the whole-plant scale, NitNRCL1 enables precise, direct, and reversible detection of NO_3_^−^ within the physiological concentration range of living plants, without inducing tissue damage or perturbing endogenous metabolic processes. This novel biosensor provides a robust methodological framework for dissecting the intricate molecular and physiological mechanisms governing plant NO_3_^−^ metabolism, as well as exploring the interplay between NO_3_^−^ utilization, environmental signaling, and plant growth regulation.

## 2. Materials and Methods

### 2.1. DNA Constructs

The construction of the sensor expression vector has been described [24]. In this study, the coding sequence of the cyanobacterial NrtA protein (PDB ID: 2G29) was optimized for plant codon usage bias and synthesized by Tsingke Biotechnology Co., Ltd. (Beijing, China). The constructs were inserted into the prokaryotic expression vector pCold II via conventional homologous recombination, harboring the 6 × Histidine tag for the purification of the recombinant fusion protein. The correct reading frame of the sensor was confirmed by sequencing analysis (Supplemental Figure S1).

### 2.2. NO_3_^−^ Detection Analysis of Purified Sensors

The amplification of the NitNRCL1 biosensor was performed in *E. coli* DH5α, and the *E. coli* Rosetta (DE3) strain was used for protein production. Cells were induced by 0.3 mM Isopropyl β-D-1-thiogalactopyranoside (IPTG) when OD_600_ reached 0.6, and proteins were expressed at 16 °C for 20 h in the Luria–Bertani (LB) medium. Thereafter, the culture was pelleted down by centrifugation at 4000× *g* for 20 min at 4 °C. The pellet was resuspended in 20 mM Tris-Cl pH 8.0 buffer for cell lysis using ultrasonication by following the reported method [25]. Biosensors were purified by metal affinity chromatography [26]. The insoluble cellular debris was cleared by centrifugation (4000× *g* for 20 min), and the cleared supernatant was subjected to Ni-NTA chromatography. Subsequently, bound protein was eluted by elution buffer (20 mM Tris-Cl and 250 mM Imidazole, pH 8.0). The eluted sensor protein was stored at 4 °C until further use.

To determine the ligand-binding specificity of the NitNRCL1 biosensor protein, the interaction of purified protein with nitrate and other related metabolites, including KCl, KNO_3_, Gly-Gly, (NH_4_)_2_SO_4_, and K_2_SO_4_ (each at 5 mM), was investigated. Experiments were performed in a 96-well plate, where 180 μL of the diluted sensor protein and 20 μL of each ligand were added to each well. After incubating the reaction system at room temperature for 5 min, 10 mM ATP and 10 μg·mL^−1^ D-luciferin potassium salt were added to initiate the luminescence reaction. Subsequently, the luminescence signal of each well was detected using a Synergy HTX multimode microplate reader (BioTek Instruments, Inc., Winooski, VT, USA), with an integration time of 1 s per well. Three technical replicates were performed for each treatment.

To analyze the binding affinity (*K*_d_) of the NitNRCL1 biosensor protein, the ligand titration curve experiment was performed [23]. This affinity assay was performed in a 96-well plate. In each well, a mixture of 180 μL of NitNRCL1 biosensor protein and 20 μL of nitrate solution with varying concentration ranges (nM to mM) were used. The luminescence signal changes in the reaction system were detected using the aforementioned method to plot the ligand titration curve and determine the binding constant. All analyses were achieved in triplicates.

### 2.3. Quantitative Detection of Nitrate in Living Cells

The NitNRCL1 biosensor was transformed into *E. coli* Rosetta (DE3). A single colony was cultured in LB medium containing 100 μg/mL ampicillin at 37 °C until OD_600_ reached 0.6. Protein expression was induced with 0.3 mM IPTG at 16 °C in the dark for 18 h. The culture was incubated at 4 °C for protein. The expressed bacterial culture was resuspended in 20 mM Tris-Cl buffer (pH 8.0) for further analysis. The luminescent signal was detected using a microplate reader according to the aforementioned method. Each well contained 180 μL of resuspended cells harboring the NitNRCL1 biosensor and 20 μL of different treatment solutions. The bacterial culture was also used for automated luminescence chemical imaging.

### 2.4. Generating Arabidopsis Transgenic Lines

The NitNRCL1 plants were generated as previously described [27,28,29]. In brief, the NitNRCL1 gene was cloned into the pCAMBIA1302 vector. Transgenic homozygous NitNRCL1 lines were obtained in the Col-0 background by floral dipping. Subsequently, PCR identification was performed using the primers detailed in Supplementary Table S1. *Arabidopsis* ecotype Columbia (Col-0) was used as the wild type (WT). Plants were grown in soil or on Petri dishes containing 1/2 MS medium at 23/20 °C, under a 16 h/8 h light/dark photoperiod, 60% RH, and a light intensity of 200 μmol m^−2^ s^−1^. Seeds were sown on soil/MS media, placed at 4 °C for 4 days in the dark, and then transferred to growth rooms. Transgenic *Arabidopsis* plants were subjected to different NO_3_^−^ deficient treatments as required before detection. Wheat and rice transformation for NitNRCL1 was performed by Biorun Bioscience Co., Ltd., Wuhan, China.

### 2.5. CCD Imaging and LUC Activity Measurement

1 mM luciferin was sprayed onto leaves, and the materials were kept in the dark for 5 min to quench the fluorescence. A low-light cooled CCD imaging apparatus (Tanon 5200, Tanon Science & Technology Co., Ltd., Shanghai, China) was used to capture the LUC image. The camera was cooled to −20 °C and relative LUC activity was measured as described. An exposure time of 3 min with 2 × 2 binning was used for all images taken [30].

### 2.6. Image Processing and Analysis

Image processing and pixel intensity were quantified using Fiji software (version 1.54f, http://fiji.sc/, accessed on 19 March 2026). Mean gray values of regions of interest (ROIs) within the root meristem region were calculated as follows: Background was subtracted from all measured intensities as generated ROIs where there was no plant material.

## 3. Results

### 3.1. Design and Optimization of NO_3_^−^ Biosensors

**Figure 1:**
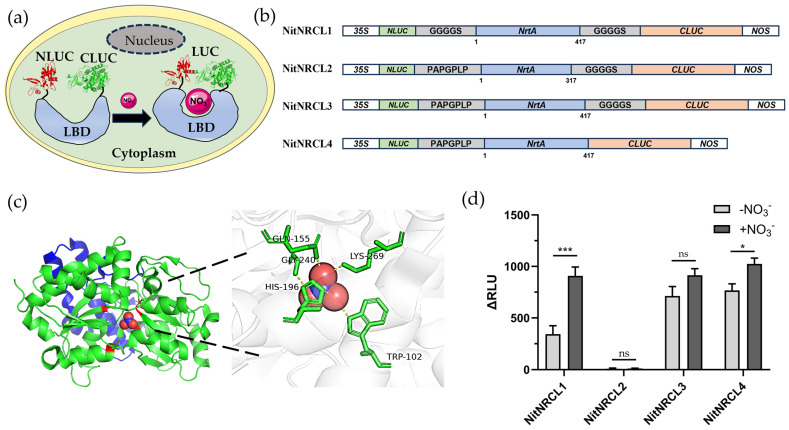
Design and Optimization of the NO_3_^−^ Biosensor NitNRCL1. (**a**) Structural model of the NO_3_^−^-bound biosensor: NO_3_^−^ binding to the ligand-binding domain (LBD) triggers a conformational shift, driving the complementation of luciferase fragments (NLuc and CLuc) and restoration of enzymatic activity. (**b**) Structural design schematics of distinct candidate NO_3_^−^ biosensors. (**c**) Three-dimensional structural model of the NrtA protein (PDB ID: 2G29), with a stick model depicting the NO_3_^−^ binding interface and key interacting residues. (**d**) Screening of candidate biosensor responsiveness to NO_3_^−^. Luciferase activity was measured as the change in relative light units (RLU) using a microplate reader before and after NO_3_^−^ supplementation. NitNRCL1 displayed the maximal induction of firefly luciferase activity. (*** *p* < 0.001, * *p* < 0.05, ns *p* ≥ 0.05, Student’s *t*-test) Error bars represent the mean ± standard deviation from *n* = 3 biological replicates.

To achieve real-time visual monitoring of NO_3_^−^ in living plants, we constructed a NO_3_^−^-specific biosensor based on the split firefly luciferase complementation system. The core design rationale of the biosensor is as follows: the NO_3_^−^ recognition domain (LBD) is inserted between split luciferase fragments (NLuc/CLuc) (Figure 1a). Upon binding to NO_3_^−^, the recognition domain undergoes a conformational shift, which drives the complementation and reconstitution of luciferase fragments, thereby restoring catalytic activity and generating a chemiluminescent signal. Members of the bacterial periplasmic binding protein (PBP) superfamily exhibit high-specificity binding affinity for small-molecule ligands, and have been exploited to develop diverse biosensors for detecting analytes including glucose [31], glutamate [32], maltose [33], glutamine [34], Zn^2+^ [35], and phosphate [36]. In this study, the cyanobacterium-derived NrtA protein was selected as the NO_3_^−^ recognition domain. Crystal structure analysis reveals that NrtA adopts a C-clamp conformation, with the NO_3_^−^ binding pocket situated between two structural domains. The oxygen atoms of NO_3_^−^ form two hydrogen bonds with Gly240, one hydrogen bond with Gln155 (2.9 Å), one hydrogen bond with Lys269 (2.8 Å), two hydrogen bonds with His196 (3.0 Å), and an additional hydrogen bond with Trp102 (2.8 Å) of NrtA (Figure 1c). Each NrtA protein specifically binds one NO_3_^−^ molecule, establishing this domain as a bona fide NO_3_^−^ binding pocket [37]. Sequence analysis further revealed that a 100-amino-acid extension at the C-terminus of NrtA (comprising an *α*-helix and two antiparallel *β*-strands) likely provides structural support for the binding pocket or participates in ligand-induced conformational rearrangements. Multiple candidate biosensors were designed with varying linker lengths and insertion positions (Figure 1b), and these constructs were screened using a prokaryotic expression system. The results demonstrated that the linker architecture is indispensable for efficient firefly luciferase reconstitution: in the presence of NO_3_^−^, the NitNRCL1 biosensor (sequence information provided in Supplemental Figure S1) exhibited the most pronounced upregulation of luciferase activity (Figure 1d). Concurrently, these findings corroborate that the C-terminal structural extension of NrtA likely stabilizes the NO_3_^−^ binding pocket and contributes to conformational dynamics associated with solute recognition and binding [38].

### 3.2. Selectivity and Kinetic Assays of the NitNRCL1

**Figure 2:**
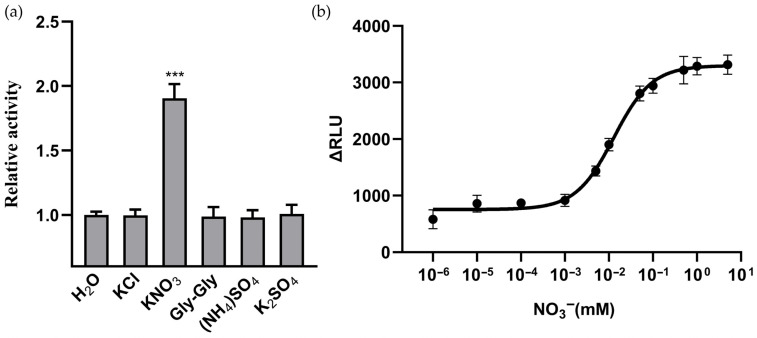
In Vitro Functional Validation of the NitNRCL1 Biosensor. (**a**) Specificity assay: Relative activity of purified NitNRCL1 protein following incubation with diverse analyte compounds. The concentrations of KCl, KNO_3_, Gly-Gly, (NH_4_)_2_SO_4_, and K_2_SO_4_ are all 5 mM. Only the NO_3_^−^ treatment groupwas significantly different from the control (*** *p* < 0.001, Student’s *t*-test). (**b**) Bioluminescence response of NitNRCL1 to increasing concentrations of NO_3_^−^, fitted to the Hill equation, *K*_d_ = 12.90 µM (*R*^2^ = 0.9816). NO_3_^−^ concentrations are indicated in the figure. Data represent the means ± SD of six biological replicates. ΔRLU, change in relative luminescence units.

To further assess the specificity of NitNRCL1 toward NO_3_^−^, purified NitNRCL1 protein was incubated with 5 mM solutions of various anionic compounds, and the luminescence intensity of the sensor protein was measured using a 96-well microplate reader, with water treatment serving as the control group. The luminescence activity of NitNRCL1 was significantly elevated in the presence of NO_3_^−^ relative to all other tested ions, confirming the high specificity of this biosensor for NO_3_^−^ (Figure 2a). Using purified NitNRCL1 protein, we determined its standardized kinetic parameters in the presence of 5 mM NO_3_^−^, and found that the response signal reached a plateau at 5 min (Figure S2). To determine the dissociation constant (*K*_d_) of NitNRCL1, changes in relative luminescence units (ΔRLU) were analyzed across a gradient of NO_3_^−^ concentrations spanning nanomolar to millimolar ranges (Figure 2b). Fitting of the resultant sigmoidal curve yielded a *K*_d_ value of approximately 12.90 μM (*R*^2^ = 0.9816), with the maximum signal response observed at 5 mM NO_3_^−^; this affinity profile is consistent with the high sensitivity of the parental NrtA protein toward NO_3_^−^ [38]. These results demonstrate that NitNRCL1 is capable of quantifying NO_3_^−^ concentrations in aqueous solutions and reporting dynamic fluctuations in NO_3_^−^ levels in real time.

### 3.3. Characterization of NitNRCL1 for NO_3_^−^ Detection in Living Prokaryotic Cells

**Figure 3:**
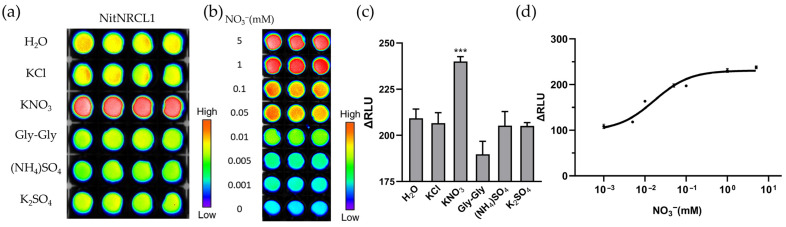
Responsiveness of NitNRCL1 Expressed in Living *E. coli* Rosetta Cells. (**a**) Specificity assessment of NitNRCL1 in living cells: Rosetta cells expressing NitNRCL1 were treated with 5 mM of the indicated compounds, and chemiluminescent signals were captured. Tested compounds are labeled in the figure. (**b**) Sensitivity assessment of NitNRCL1 in living cells: Rosetta cells expressing NitNRCL1 were treated with gradient concentrations of NO_3_^−^, and luminescence signals were recorded. NO_3_^−^ concentrations are indicated in the figure. (**c**) Statistical analysis of relative luminescence activity changes induced by the indicated compounds in panel. Only the NO_3_^−^ treatment group was significantly different from the control (*** *p* < 0.001, Student’s *t*-test). (**d**) Dose-response curve of ΔRLU values from panel B, fitted to the Hill equation. All values represent the means ± standard deviations (SD) of three biological replicates (*n* = 3). *K*_d_ = 16.69µM, R^2^ = 0.9674. ΔRLU, change in relative luminescence units.

To validate the specificity and concentration-dependent responsiveness of NitNRCL1 toward NO_3_^−^ in living prokaryotic cells, the biosensor was heterologously expressed in *Escherichia coli* Rosetta (DE3) cells. Chemiluminescent imaging was performed on Rosetta cell suspensions expressing NitNRCL1 following treatment with 5 mM solutions of various test compounds, confirming that the biosensor retains strict specificity for NO_3_^−^ in the intracellular environment of living bacteria (Figure 3a). In addition, we found that live cells expressing NitNRCL1 showed no obvious response to nitrite (Figure S3). Statistical analysis of relative luminescence activity revealed a highly significant signal response in the NO_3_^−^-treated group compared with all other compound treatments (Figure 3c). Exposure of NitNRCL1-expressing Rosetta cells to increasing concentrations of NO_3_^−^ resulted in a corresponding dose-dependent increase in luminescence signal intensity (Figure 3b). Quantification of ΔRLU values from Figure 3b further demonstrated a strong positive correlation between NO_3_^−^ concentration and luminescence intensity in bacterial suspensions, with signal intensity gradually escalating alongside rising NO_3_^−^ levels (Figure 3d). Collectively, these assays verify that NitNRCL1 can specifically bind to intracellular NO_3_^−^ and enable quantitative detection of NO_3_^−^ in living cells.

### 3.4. Expression and NO_3_^−^ Responsiveness of the Biosensor in Plants

**Figure 4:**
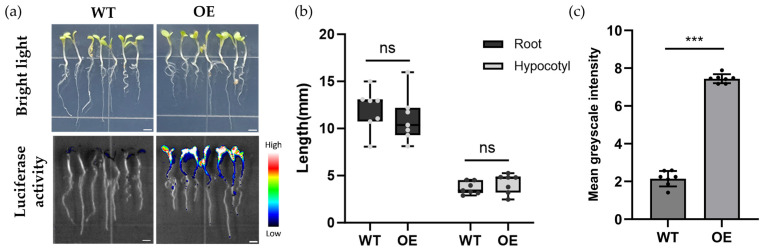
Expression of NitNRCL1 in Transgenic Plants. (**a**) Phenotypic analysis (upper panel) of 7-day-old wild-type (Col-0) and transgenic *Arabidopsis* (OE) seedlings grown on 1/2 MS medium, and corresponding chemiluminescent signals (lower panel) induced by 10 mM NO_3_^−^ treatment. Scale bar, 2 mm. (**b**) Statistical analysis of root and hypocotyl lengths of 7-day-old *Arabidopsis* seedlings; ns indicates no significant difference (*p* ≥ 0.05). (**c**) Statistical analysis of the mean gray values of chemiluminescent signals in 7-day-old *Arabidopsis* seedlings. Data represent the means ± SD (*n* = 7, *** *p* < 0.001, Student’s *t*-test).

To evaluate the expression efficiency and functionality of NitNRCL1 in plant cells, *Arabidopsis thaliana*, tobacco leaves, and carrot callus stably transformed with NitNRCL1 were treated with NO_3_^−^. Results confirmed that NitNRCL1 is fully expressed and retains NO_3_^−^ responsiveness in diverse plant systems (Figure 4a, Figures S4 and S5). We identified 10 independent NitNRCL1 transgenic *Arabidopsis* lines (Figure S6), selected four of them for absolute quantitative qPCR analysis (Figure S7), and used these lines to verify the function of NitNRCL1 in *Arabidopsis*. All four lines showed a good response to NO_3_^−^ (Figure S8). To establish a stable platform for in planta NO_3_^−^ monitoring, transgenic *Arabidopsis* lines constitutively expressing NitNRCL1 (OE) were generated via *Agrobacterium*-mediated transformation. No significant differences in root length, hypocotyl length, or overall vegetative phenotype were observed between OE lines and wild-type (Col-0) plants (Figure 4b), indicating that biosensor expression does not perturb normal plant growth and development. Chemiluminescent detection revealed robust, stable luminescence signals in transgenic *Arabidopsis* seedlings grown on nitrogen-replete medium, whereas no detectable signal was observed in wild-type plants (Figure 4c), verifying stable expression and strong NO_3_^−^ responsiveness of the biosensor in transgenic plants.

### 3.5. Functional Validation of NitNRCL1 in Plants

**Figure 5:**
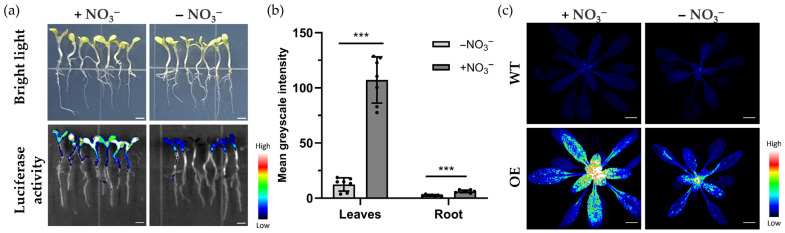
Functional Validation of NitNRCL1 in Transgenic *Arabidopsis thaliana*. (**a**) Chemiluminescent imaging of OE *Arabidopsis* seedlings under NO_3_^−^-replete (10 mM KNO_3_) and NO_3_^−^-deprived conditions. Scale bar, 2 mm. (**b**) Statistical analysis of the mean gray values in leaves and roots. All values represent the means ± SD (*n* = 7; *** *p* < 0.001, Student’s *t*-test). (**c**) Chemiluminescent imaging of wild-type and OE *Arabidopsis* under NO_3_^−^-replete and NO_3_^−^-deprived conditions. Scale bar, 10 mm.

To characterize the NO_3_^−^ responsiveness of NitNRCL1 in transgenic plants, OE *Arabidopsis* seedlings were subjected to differential nitrogen treatments. Seedlings grown on NO_3_^−^-supplemented medium for 7 days exhibited intense, widespread chemiluminescent signals in aerial tissues following 10 mM KNO_3_ treatment for 10 min, whereas only faint, localized signals were detected in the NO_3_^−^-deprived control group (Figure 5a). Gray value quantification confirmed that signal intensity was significantly higher in both leaves and roots of the NO_3_^−^-treated group relative to the control, with leaf signals exceeding those in roots (Figure 5b). To assess the broad applicability of NitNRCL1 across crop species, the biosensor was heterologously expressed and functionally validated in wheat and rice, demonstrating reliable NO_3_^−^ detection capacity in these monocot crops (Figure S9). Subcellular localization of NitNRCL1 in wheat protoplasts revealed that it is localized in the cytoplasm (Figure S10). Furthermore, analysis of 21-day-old soil-grown plants revealed no detectable luminescence signals in wild-type *Arabidopsis* under either NO_3_^−^-replete or NO_3_^−^-deprived conditions. In contrast, OE plants displayed intense, uniform chemiluminescent signals in leaves under NO_3_^−^ supplementation, with significantly stronger signals in young leaves relative to mature leaves. Under NO_3_^−^ deprivation, leaf signals were drastically attenuated, with only faint signals retained in young tissues (Figure 5c). These findings confirm that NitNRCL1 specifically responds to NO_3_^−^ across distinct developmental stages in plants, with signal distribution closely aligning with the tissue-specific allocation patterns of endogenous NO_3_^−^ [39], establishing a robust tool for in vivo monitoring of plant nitrogen status.

### 3.6. Dynamic Monitoring of Nitrate Uptake and Translocation in Plants Under Low-Nitrogen Stress

**Figure 6:**
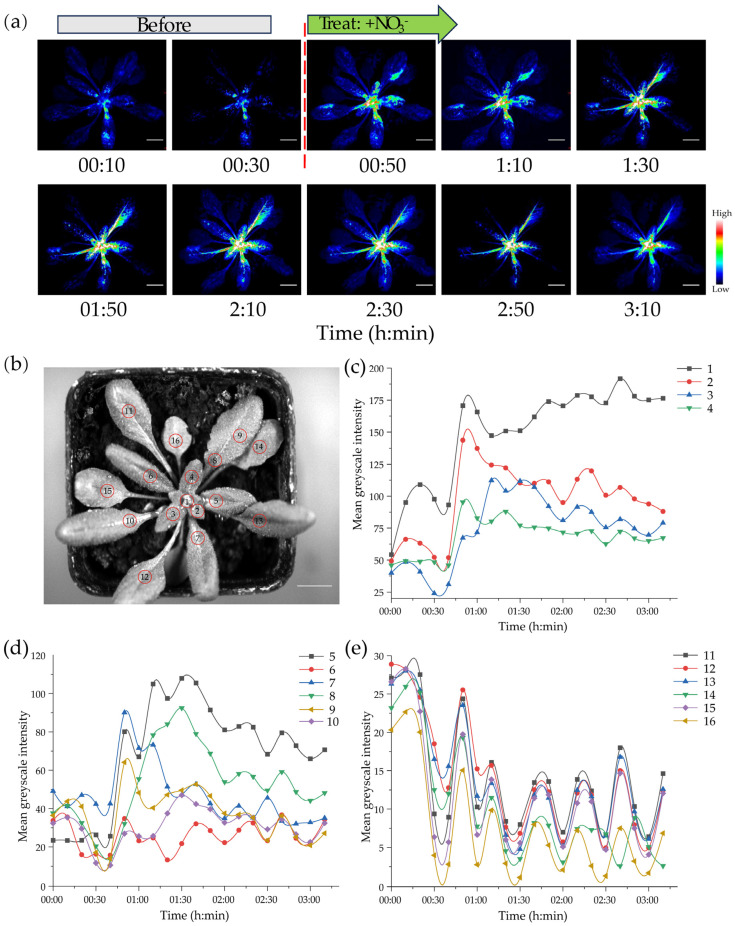
Dynamic Monitoring of NO_3_^−^ Uptake and Translocation in Transgenic *Arabidopsis* via NitNRCL1. (**a**) In vivo live imaging tracking of NO_3_^−^ signals in OE *Arabidopsis* seedlings. Following 7 days of nitrogen starvation, continuous luminescence monitoring was initiated 30 min prior to treatment with 10 mM KNO_3_ (+NO_3_^−^), with signal dynamics recorded post-treatment. Time points are denoted in h:min. Before, pre-NO_3_^−^ treatment; +NO_3_^−^, post-NO_3_^−^ treatment. Scale bar, 10 mm. (**b**) Schematic of 16 marked leaf zones of interest (red circles) for spatial quantitative gray value analysis, with zones 1–4 designated as young central leaves, zones 5–10 as expanding leaves, and zones 11–16 as mature peripheral leaves. Scale bar, 10 mm. (**c**–**e**) Time-mean gray value curves depicting luminescence intensity changes in each marked ROI over time, with the timing of NO_3_^−^ application indicated.

To evaluate the suitability of NitNRCL1 for spatiotemporal monitoring of NO_3_^−^ distribution in living plants, 14-day-old OE *Arabidopsis* seedlings were subjected to 7 days of nitrogen starvation, followed by in vivo live imaging to track the dynamic uptake and translocation of NO_3_^−^ in aerial tissues. Nitrogen-starved OE plants exhibited weak, spatially restricted luminescence signals prior to NO_3_^−^ resupply. Upon treatment with 10 mM KNO_3_ (+NO_3_^−^), luminescence intensity increased dramatically over time, accompanied by a distinct, orderly signal propagation pattern (Figure 6a). Initial signals emerged exclusively in the shoot apical meristem, young leaves, and vascular veins—representing high-intensity signals in young conductive tissues—reflecting the physiological priority of NO_3_^−^ allocation to developing tissues [40]. Subsequently, NO_3_^−^ signals spread rapidly along the leaf vascular system and gradually extended into mature leaf tissues, forming a centripetal gradient distribution consistent with long-distance translocation routes mediated by the vascular network [39]. After 2 h of NO_3_^−^ treatment, signals stabilized across all leaf tissues but retained clear tissue specificity, with significantly higher intensity in young leaves than mature leaves, indicative of differential NO_3_^−^ partitioning between tissue types. This dynamic pattern aligns perfectly with the physiological mechanisms of NO_3_^−^ transport in plants, demonstrating that NitNRCL1 enables high-spatiotemporal-resolution tracking of the entire NO_3_^−^ uptake-to-translocation cascade.

To quantify temporal changes in NO_3_^−^ levels across nitrogen-starved OE *Arabidopsis* discrete leaf regions, 16 zones of interest (ROIs) were marked on seedling leaves (Figure 6b), and time-intensity curves of luminescence signals were generated for each ROI (Figure 6c–e). Following NO_3_^−^ resupply, luminescence signals in young leaf zones (1–4) peaked rapidly within 10 min, whereas peak signal occurrence in mature leaf zones (11–16) was markedly delayed by 30–60 min. The variation in leaves 5–10 is intermediate between that of juvenile and mature leaves. Quantitative tracing of NO_3_^−^ signals in planta partially reflects the source-to-sink allocation pattern of nitrogen nutrients in plants [41]. Existing literature confirms that long-distance NO_3_^−^ transport in plants is predominantly mediated by the xylem vascular system [42,43,44], a mechanism fully consistent with the dynamic signal propagation patterns observed with NitNRCL1. All ROIs exhibited consistent kinetic trends, further validating NitNRCL1 as a reliable tool for quantitative and real-time tracing of endogenous NO_3_^−^ in plants. Gray value-based quantitative analysis enables precise kinetic monitoring of NO_3_^−^ translocation, providing a powerful asset for studying plant nitrogen dynamics. To verify whether NitNRCL1 transgenic plants can reversibly detect NO_3_^−^, we sprayed leaves of NitNRCL1 transgenic *Arabidopsis* plants with 10 mM NO_3_^−^ for reversible NO_3_^−^ detection assays. The results showed that NitNRCL1 exhibited good repeatable detection characteristics (Figure S11).

## 4. Discussion

As a core macronutrient and pivotal signaling molecule governing plant growth and development, the spatiotemporal dynamic allocation of NO_3_^−^ is a central focus for elucidating nitrogen use efficiency in plants. Conventional detection methods are limited by destructive sampling or localized monitoring, failing to meet the demand for real-time, whole-plant dynamic tracking of endogenous NO_3_^−^. Through multi-system validation in prokaryotic and eukaryotic hosts, this study demonstrates that the split-luciferase biosensor NitNRCL1, engineered using the cyanobacterial NrtA protein, achieves accurate, specific, and dynamic tracing of NO_3_^−^ in planta. This novel tool opens new avenues for dissecting the complex regulatory networks underlying NO_3_^−^ uptake, translocation, and assimilation in plant nitrogen metabolism.

In recent years, FRET-based fluorescent biosensors have revolutionized ion monitoring in plants, with several NO_3_^−^ sensors developed including NitraMeter3.0 [45], mCitrine-NLP7 [46], FLIP-NT [37], NiTrac-NPF1.3 [47], and NiTrac1 [48]; these tools offer exceptional spatial resolution and non-invasive measurement capabilities. Compared with these existing sensors, the defining advantage of NitNRCL1 lies in its capacity for whole-plant dynamic NO_3_^−^ monitoring. Compared with these existing sensors, NitNRCL1 in this study not only maintains high spatial resolution and non-invasive detection but also enables dynamic monitoring of whole-plant NO_3_^−^ levels, allowing simultaneous multi-site assessment of differential NO_3_^−^ uptake and translocation across distinct organs and tissues. As demonstrated by dynamic tracking of NO_3_^−^ in NitNRCL1 transgenic *Arabidopsis* following nitrogen deprivation, the NO_3_^−^ signal initially accumulates in young developing leaves and vascular conducting tissues [40,45,46,47,48,49] and subsequently radiates from leaf veins into the surrounding mesophyll cells [50,51], thereby reflecting, to a certain extent, the transport and partitioning patterns of nitrogen nutrients within the plant [41,42,43,44]. This capability forms a robust complement to FRET-based sensors that operate at cellular or subcellular resolution. A detailed performance comparison of NitNRCL with existing NO_3_^−^ biosensors is available in Supplementary Data (Table S2).

Functional assays confirm that NitNRCL1 is stably expressed and faithfully responds to NO_3_^−^ across all developmental stages of *Arabidopsis*, without inducing abnormal phenotypic alterations in transgenic plants. Critically, the chemiluminescent output of NitNRCL1 eliminates the need for complex fluorescence microscopy equipment, making it uniquely suited for whole-plant macroscopic imaging and high-throughput screening. This feature positions NitNRCL1 as a precision tool for functional validation of nitrogen-use-efficiency genes and accelerated crop molecular breeding.

Despite its exceptional application potential, NitNRCL1 has avenues for further optimization. The current sensor response time (approximately 10 min to peak signal) precludes capture of the rapid, second-scale transient responses mediated by nitrate transporters. Notably, wild-type plants were used as background controls in this study; however, the lack of mechanistic controls based on mutants unable to bind NO_3_^−^ imposes limitations on demonstrating that the observed signal specifically depends on the interaction of NO_3_^−^ with the NrtA domain. Additionally, heterologous expression efficiency and signal stability in monocot crops such as rice and wheat require enhancement via codon optimization and replacement with crop-specific promoters. Future directions include directed evolution of the NrtA protein to accelerate conformational switching, integration with other independent detection techniques to enable absolute quantification of nitrate concentrations, fusion of subcellular localization signals to generate organelle-specific NO_3_^−^ sensors, and integration of multi-biosensor designs to enable simultaneous monitoring of NO_3_^−^ with calcium, cytokinins, and other signaling molecules. Furthermore, coupling NitNRCL1 with near-infrared luciferase technology to develop portable detection devices will facilitate the translation of precise NO_3_^−^ tracing from laboratory research to field-scale in situ monitoring, providing critical technical support for precision fertilization and sustainable agricultural development.

## 5. Conclusions

In summary, this study successfully developed a split-luciferase-based genetically encoded NO_3_^−^ biosensor, NitNRCL1. Multi-level validation in prokaryotic and eukaryotic systems confirms that NitNRCL1 exhibits high specificity, a broad concentration-response range, and excellent biocompatibility, enabling real-time, whole-plant dynamic monitoring of NO_3_^−^ in *Arabidopsis*. This biosensor not only provides a novel platform for dissecting the molecular mechanisms governing NO_3_^−^ uptake, translocation, and assimilation, but also delivers technical support for the development of nitrogen-use-efficient crop varieties and precision fertilization strategies. Future optimization of NitNRCL1—including enhanced NO_3_^−^ responsiveness, crop-specific engineering, and multi-signal integration—will further expand its utility in plant nitrogen metabolism research and sustainable agriculture, offering innovative solutions to mitigate nitrogen pollution and improve crop productivity.

## Data Availability

All data needed to evaluate the conclusions in the paper are present in the paper and/or the Supplementary Materials. Further inquiries can be directed to the corresponding authors.

## Supplementary Materials

The following supporting information can be downloaded at: https://www.mdpi.com/article/10.3390/bios16050243/s1, This PDF file includes: Figure S1: Amino acid sequence of the NitNRCL1 sensor; Figure S2: In vitro kinetic characterization of the NitNRCL1 sensor; Figure S3: Response of the NitNRCL1 sensor to nitrate and nitrite in living *Escherichia coli* cells; Figure S4: PCR identification of the NitNRCL1 sensor gene in diverse plant materials; Figure S5: Functional validation of the NitNRCL1 sensor in diverse plant materials; Figure S6: PCR identification of positive transgenic *Arabidopsis* thaliana plants; Figure S7: Absolute quantification of transcripts encoding the NitNRCL1 sensor in transgenic *Arabidopsis* thaliana overexpression lines; Figure S8: Response of transgenic Arabidopsis thaliana lines with varying expression levels to nitrate; Figure S9: Functional validation of the NitNRCL1 sensor in rice and wheat; Figure S10: Subcellular localization of NitNRCL1 in wheat protoplasts; Figure S11: Reversibility of nitrate detection in transgenic *Arabidopsis* thaliana plants expressing NitNRCL1; Table S1: List of primers used in this study; Table S2: Comparison of NitNRCL1 with existing nitrate biosensors.

## Abbreviations

The following abbreviations are used in this manuscript:

IPTG	Isopropyl β-D-1-thiogalactopyranoside
LBD	Ligand-binding domain
NLuc	N-terminal luciferase fragment
CLuc	C-terminal luciferase fragment
PBP	Periplasmic binding protein
*K*_d_	Dissociation constant
ΔRLU	Change in relative luminescence units
OD_600_	Optical density at 600 nm
Ni-NTA	Nickel-nitrilotriacetic acid
WT	Wild type
OE	NitNRCL1 overexpression lines in *Arabidopsis*
Col-0	Columbia-0
MS	Murashige and Skoog medium
CCD	Charge-coupled device
ROI	Region of interest
K*_m_*	Michaelis constant

## References

[1] Liu X., Hu B., Chu C. (2022). Nitrogen assimilation in plants: Current status and future prospects. J. Genet. Genom..

[2] Fredes I., Moreno S., Diaz F.P., Gutierrez R.A. (2019). Nitrate signaling and the control of *Arabidopsis* growth and development. Curr. Opin. Plant Biol..

[3] Gao Y., Qi S., Wang Y. (2022). Nitrate signaling and use efficiency in crops. Plant Commun..

[4] Ho C.H., Lin S.H., Hu H.C., Tsay Y.F. (2009). CHL1 functions as a nitrate sensor in plants. Cell.

[5] Sakakibara H. (2021). Cytokinin biosynthesis and transport for systemic nitrogen signaling. Plant J..

[6] Liu K.-H., Niu Y., Konishi M., Wu Y., Du H., Chung Hoo S., Li L., Boudsocq M., McCormack M., Maekawa S. (2017). Discovery of nitrate-CPK-NLP signalling in central nutrient-growth networks. Nature.

[7] Vidal E.A., Alvarez J.M., Araus V., Riveras E., Brooks M.D., Krouk G., Ruffel S., Lejay L., Crawford N.M., Coruzzi G.M. (2020). Nitrate in 2020: Thirty years from transport to signaling networks. Plant Cell.

[8] Xuan W., Beeckman T., Xu G. (2017). Plant nitrogen nutrition: Sensing and signaling. Curr. Opin. Plant Biol..

[9] Zhang G.B., Meng S., Gong J.M. (2018). The expected and unexpected roles of nitrate transporters in plant Abiotic Stress Resistance and Their Regulation. Int. J. Mol. Sci..

[10] Parker J.L., Newstead S. (2014). Molecular basis of nitrate uptake by the plant nitrate transporter NRT1.1. Nature.

[11] Orsel M., Chopin F., Leleu O., Smith S.J., Krapp A., Daniel-Vedele F., Miller A.J. (2006). Characterization of a two-component high-affinity nitrate uptake system in *Arabidopsis*. Physiology and protein-protein interaction. Plant Physiol..

[12] Ota R., Ohkubo Y., Yamashita Y., Ogawa-Ohnishi M., Matsubayashi Y. (2020). Shoot-to-root mobile CEPD-like 2 integrates shoot nitrogen status to systemically regulate nitrate uptake in *Arabidopsis*. Nat. Commun..

[13] Ball S., Colleoni C., Cenci U., Raj J.N., Tirtiaux C. (2011). The evolution of glycogen and starch metabolism in eukaryotes gives molecular clues to understand the establishment of plastid endosymbiosis. J Exp Bot..

[14] Demes E., Besse L., Cubero-Font P., Satiat-Jeunemaitre B., Thomine S., De Angeli A. (2020). Dynamic measurement of cytosolic pH and [NO_3_^−^] uncovers the role of the vacuolar transporter AtCLCa in cytosolic pH homeostasis. Proc. Natl. Acad. Sci. USA.

[15] Zhen R.G., Koyro H.W., Leigh R.A., Tomos A.D., Miller A.J. (1991). Compartmental nitrate concentrations in barley root cells measured with nitrate-selective microelectrodes and by single-cell sap sampling. Planta.

[16] Zhang C., Demes-Causse E., Li X., Guo Z., Zhao H., Cao H., Song Y., Mu L., Zhang K., Zhang J. (2026). The Maize ZmbHLH118 transcription factor regulates vacuolar nitrate loading by the NO_3_^−^ transporter ZmCLCa. Adv. Sci..

[17] Mooshammer M., Wanek W., Hämmerle I., Fuchslueger L., Hofhansl F., Knoltsch A., Schnecker J., Takriti M., Watzka M., Wild B. (2014). Adjustment of microbial nitrogen use efficiency to carbon: Nitrogen imbalances regulates soil nitrogen cycling. Nat. Commun..

[18] Green L.C., Wagner D.A., Glogowski J., Skipper P.L., Wishnok J.S., Tannenbaum S.R. (1982). Analysis of nitrate, nitrite, and [^15^N] nitrate in biological fluids. Anal. Biochem..

[19] Hao C.K., Dungait J.A.J., Shang W.H., Hou R.X., Gong H.R., Yang Y.F., Lambers H., Yu P., Delgado-Baquerizo M., Xu X.L. (2025). Conservation agriculture raises crop nitrogen acquisition by amplifying plant-microbe synergy under climate warming. Nat. Commun..

[20] Cookson S.J., Williams L.E., Miller A.J. (2005). Light-dark changes in cytosolic nitrate pools depend on nitrate reductase activity in *Arabidopsis* leaf cells. Plant Physiol..

[21] Henriksen G.H., Raman D.R., Walker L.P., Spanswick R.M. (1992). Measurement of Net Fluxes of ammonium and nitrate at the surface of barley roots using ion-selective microelectrodes: II. Patterns of uptake along the root axis and evaluation of the microelectrode Flux estimation technique. Plant Physiol..

[22] Woo S.G., Moon S.J., Kim S.K., Kim T.H., Lim H.S., Yeon G.H., Sung B.H., Lee C.H., Lee S.G., Hwang J.H. (2020). A designed whole-cell biosensor for live diagnosis of gut inflammation through nitrate sensing. Biosens. Bioelectron..

[23] Taneoka A., Sakaguchi-Mikami A., Yamazaki T., Tsugawa W., Sode K. (2009). The construction of a glucose-sensing luciferase. Biosens. Bioelectron..

[24] Jones A.M., Grossmann G., Danielson J., Sosso D., Chen L.Q., Ho C.H., Frommer W.B. (2013). In vivo biochemistry: Applications for small molecule biosensors in plant biology. Curr. Opin. Plant Biol..

[25] Khan P., Prakash A., Hague M.A., Islam A., Hassan M.I., Ahmad F. (2016). Structural basis of urea-induced unfolding: Unraveling the folding pathway of hemochromatosis factor E. Int. J. Biol. Macromol..

[26] Kielkopf C.L., Bauer W., Urbatsch I.L. (2020). Purification of polyhistidine-tagged proteins by immobilized metal affinity chromatography. Cold Spring Harb. Protoc..

[27] Ursache R., Fujita S., Dénervaud Tendon V., Geldner N. (2021). Combined fluorescent seed selection and multiplex CRISPR/Cas9 assembly for fast generation of multiple *Arabidopsis* mutants. Plant Methods.

[28] Sparkes I.A., Runions J., Kearns A., Hawes C. (2006). Rapid, transient expression of fluorescent fusion proteins in tobacco plants and generation of stably transformed plants. Nat. Protoc..

[29] Liu M.S., Huang T.K., Wang Y.C., Wang S.C., Wu C.H., Kuo C.H., Lai E.M. (2026). Floral stage optimization and immune evasion enhance Agrobacterium-mediated genome editing in *Arabidopsis*. New Phytol..

[30] Chen H.M., Zou Y., Shang Y.L., Lin H.Q., Wang Y.J., Cai R., Tang X.Y., Zhou J.M. (2008). Firefly luciferase complementation imaging assay for protein-protein interactions in plants. Plant Physiol..

[31] Wang J., Carmon K.S., Luck L.A., Suni I.I. (2005). Electrochemical impedance biosensor for glucose detection utilizing a periplasmic *E. coli* receptor protein. Electrochem. Solid-State Lett..

[32] Okumoto S., Looger L.L., Micheva K.D., Reimer R.J., Smith S.J., Frommer W.B. (2005). Detection of glutamate release from neurons by genetically encoded surface-displayed FRET nanosensors. Proc. Natl. Acad. Sci. USA.

[33] Gilardi G., Zhou L.Q., Hibbert L., Cass A.E. (1994). Engineering the maltose binding protein for reagentless fluorescence sensing. Anal. Chem..

[34] Wada A., Mie M., Aizawa M., Lahoud P., Cass A.E.G., Kobatake E. (2003). Design and construction of glutamine binding proteins with a self-adhering capability to unmodified hydrophobic surfaces as reagentless fluorescence sensing devices. J. Am. Chem. Soc..

[35] Dwyer M.A., Looger L.L., Hellinga H.W. (2003). Computational design of a Zn^2+^ receptor that controls bacterial gene expression. Proc. Natl. Acad. Sci. USA.

[36] Salins L.L., Deo S.K., Daunert S. (2004). Phosphate binding protein as the biorecognition element in a biosensor for phosphate. Sens. Actuators B Chem..

[37] Fatima U., Ameen F., Soleja N., Khan P., Almansob A., Ahmad A. (2020). A fluorescence resonance energy transfer-based analytical tool for nitrate quantification in Living Cells. ACS Omega.

[38] Koropatkin N.M., Pakrasi H.B., Smith T.J. (2006). Atomic structure of a nitrate-binding protein crucial for photosynthetic productivity. Proc. Natl. Acad. Sci. USA.

[39] Ruffel S., Del Rosario J., Lacombe B., Rouached H., Gutiérrez R.A., Coruzzi G.M., Krouk G. (2025). Nitrate sensing and signaling in plants: Comparative insights and nutritional interactions. Annu. Rev. Plant Biol..

[40] Kiba T., Feria-Bourrellier A.B., Lafouge F., Lezhneva L., Boutet-Mercey S., Orsel M., Bréhaut V., Miller A., Daniel-Vedele F., Sakakibara H. (2012). The *Arabidopsis* nitrate transporter NRT2.4 plays a double role in roots and shoots of nitrogen-starved plants. Plant Cell.

[41] Drechsler N., Zheng Y., Bohner A., Nobmann B., von Wirén N., Kunze R., Rausch C. (2015). Nitrate-dependent control of shoot K homeostasis by the nitrate transporter1/peptide transporter family member NPF7.3/NRT1.5 and the stelar K^+^ outward rectifier SKOR in *Arabidopsis*. Plant Physiol..

[42] Lin S.H., Kuo H.F., Canivenc G., Lin C.S., Lepetit M., Hsu P.K., Tillard P., Lin H.L., Wang Y.Y., Tsai C.B. (2008). Mutation of the *Arabidopsis NRT1.5* nitrate transporter causes defective root-to-shoot nitrate transport. Plant Cell.

[43] Aluko O.O., Kant S., Adedire O.M., Li C.Z., Yuan G., Liu H.B., Wang Q. (2023). Unlocking the potentials of nitrate transporters at improving plant nitrogen use efficiency. Front. Plant Sci..

[44] Chen K.E., Chen H.Y., Tseng C.S., Tsay Y.F. (2020). Improving nitrogen use efficiency by manipulating nitrate remobilization in plants. Nat. Plants.

[45] Chen Y.N., Cartwright H.N., Ho C.H. (2022). In vivo visualization of nitrate dynamics using a genetically encoded fluorescent biosensor. Sci. Adv..

[46] Liu K.H., Liu M., Lin Z., Wang Z.-F., Chen B., Liu C., Guo A., Konishi M., Yanagisawa S., Wagner G. (2022). NIN-like protein 7 transcription factor is a plant nitrate sensor. Science.

[47] Chen Y.N., Ho C.H. (2022). Concept of Fluorescent Transport activity biosensor for the characterization of the *Arabidopsis* NPF1.3 Activity of Nitrate. Sensors.

[48] Ho C.H., Frommer W.B. (2014). Fluorescent sensors for activity and regulation of the nitrate transceptor CHL1/NRT1.1 and oligopeptide transporters. eLife.

[49] Tang Z., Fan X., Li Q., Feng H., Miller A.J., Shen Q., Xu G. (2012). Knockdown of a Rice stelar nitrate transporter alters long-distance translocation but not root influx. Plant Physiol..

[50] Fan S.C., Lin C.S., Hsu P.K., Lin S.H., Tsay Y.F. (2009). The *Arabidopsis* nitrate transporter NRT1.7, expressed in phloem, is responsible for source-to-sink remobilization of nitrate. Plant Cell.

[51] Li Y.G., Ouyang J., Wang Y.Y., Hu R., Xia K.F., Duan J., Wang Y.Q., Tsay Y.F., Zhang M.Y. (2015). Disruption of the rice nitrate transporter OsNPF2.2 hinders root-to-shoot nitrate transport and vascular development. Sci. Rep..

