# Supplementary material for: Genetically Encoded Fluorescent Biosensors Enable Noninvasive Real-Time Visualization of Nitrate Dynamics in Intact Living Plants
Source: Biosensors (Basel). 2026 Apr 26;16(5):243. doi: 10.3390/bios16050243 (PMC13204499; doi:10.3390/bios16050243)
Supplement: Supplementary file 1 [file biosensors-16-00243-s001.zip › biosensors-4241326-supplementary.pdf]

biosensors

MDPI

Supplementary

Genetically Encoded Fluorescent Biosensors Enable Noninvasive Real-Time Visualization of Nitrate Dynamics in Intact Living Plants

Li Zhang [1], Qing Xu [1], Changxu Wang [1], Jinfeng Wang [1], Jing Yue [1], Yin Lu [1], Guangle Zhang [1], Lixue Yuan [1], Yonghua Wang [1,2,3], Bo Yu [1,2,3,*] and Guozhang Kang [1,2,3,*]

[1] The National Engineering Research Center for Wheat, Henan Agricultural University, Zhengzhou 450046, China; zhangli@stu.henau.edu.cn (L.Z.); xuqing@stu.henau.edu.cn (Q.X.); wangchangxu@stu.henau.edu.cn (C.W.); jinfengwang@stu.henau.edu.cn (J.W.); yuejing@stu.henau.edu.cn (J.Y.); luyin@stu.henau.edu.cn (Y.L.); zhangguangle@stu.henau.edu.cn (G.Z.); yuanlixue@stu.henau.edu.cn (L.Y.); wangyonghua@henau.edu.cn (Y.W.)

[2] The State Key Laboratory of High-Efficiency Production of Wheat-Maize Double Cropping, Henan Agricultural University, Zhengzhou 450046, China

[3] Functional Crop Engineering Center in Henan Province, Henan Agricultural University, Zhengzhou 450046, China

* Correspondence: yubo@henau.edu.cn (B.Y.); guozhangkang@henau.edu.cn (G.K.)

```
MSGYVNNPEATNALIDKDGWLHSGDIAYWDEDEHFFIVDRLKSLIKYKGYQVAPAELESILLQHPNIFDAGVAGLPDDDA
GELPAAVVVLEHGKTMTEKEIVDYVASQVTTAKKLRGGVVFVDEVPKGLTGKLDARKIREILIKAGGGGSGTGSSTDQAI
SPLVEGENAPEVTTAKLGFIALTDAAPLIIAKEKGFYAKYGMPDVEVLKQASWGTTRDNLVLGSASGGIDGAHILTPMPY
LITMGTVTDGKPTPMYILARLNVNGQGIQLGNNYKDLKVGTDAAPLKEAFAKVTDPKVAMTFPGGTHDMWIRYWLAAGGM
EPGKDFSTIVVPPAQMVANVKVNAMESFCVGEPWPLQTVNQGVGYQALTTGQLWKDHPEKAFGMRADWVDQNPKAAKALL
MAVMEAQQWCDQAENKEEMCQILSKREWFKVPFEDIIDRSKGIYNFGNGQETFEDQEIMQKYWVDNASYPYKSHDQWFLT
ENIRWGYLPASTDTKAIVDKVNREDLWREAAQALEVPADQIPSSPSRGIETFFDGITFDPENPQAYLDSLKIKSIKAGGG
GSEDAKNIKKGPAPFYPLEDGTAGEQLHKAMKRYALVPGTIAFTDAHIEVDITYAEYFEMSVRLAEAMKRYGLNTNHRIV
VCSENSLQFFMPVLGALFIGVAVAPANDIYNERELLNSMGISQPTVVFVSKKGLQKILNVQKKLPIIQKIIIMDSKTDYQ
GFQSMYTFVTSHLPPGFNEYDFVPESFDRDKTIALIMNSSGSTGLPKGVALPHRTACVRFSHARDPIFGNQIIPDTAILS
VVPFHHGFGMFTTLGYLICGFRVVLMYRFEEELFLRSLQDYKIQSALLVPTLFSFFAKSTLIDKYDLSNLHEIASGGAPL
SKEVGEAVAKRFHLPGIRQGYGLTETTSAILITPEGDDKPGAVGKVVPFFEAKVVDLDTGKTLGVNQRGELCVRGPMIMS
GYVNNPEATNALIDKDG
```

Figure S1. Amino acid sequence of the NitNRCL1 sensor.

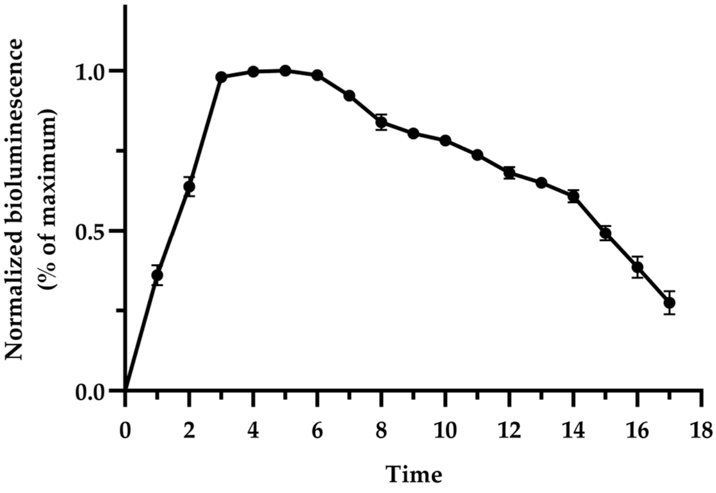

Figure S2. In vitro kinetic characterization of the NitNRCL1 sensor. The temporal bioluminescence response of purified NitNRCL1 protein was measured using a microplate reader following the addition of substrate and 5 mM NO_3^-. Normalized bioluminescence signals are presented as mean ± standard deviation (n = 4 independent replicates). The ascending phase was fitted using a one-phase association model (baseline constrained to 0), yielding a half-life of 1.35 minutes (95% confidence interval: 1.15–1.75) ($R^2 = 0.965$). The descending phase was fitted using a one-phase exponential decay model (plateau constrained to 0), yielding a half-life of 10.1 minutes (95% confidence interval: 9.16–11.2) ($R^2 = 0.893$).

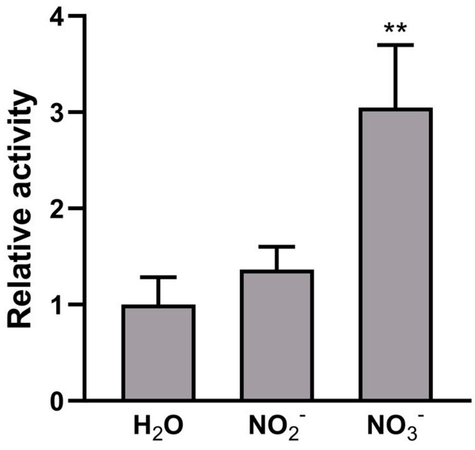

Figure S3. Response of the NitNRCL1 sensor to nitrate and nitrite in living *Escherichia coli* cells. Living E. coli cells expressing NitNRCL1 were treated with deionized water (H_2O, negative control), 5 mM nitrite (NO_2^-), or 5 mM nitrate (NO_3^-), after which luminescence signals (relative light units, RLU) were measured using a microplate reader. Data are presented as mean ± standard deviation (n = 4 independent biological replicates) (**$p < 0.01$). These results demonstrate that the sensor exhibits a robust response to nitrate but shows no appreciable response to nitrite.

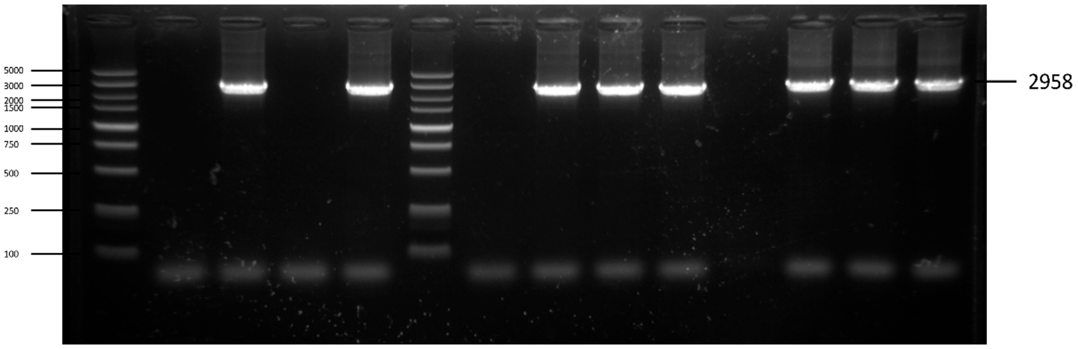

Figure S4. PCR identification of the NitNRCL1 sensor gene in diverse plant materials. No target bands were detected in the uninoculated/wild-type (WT) *Nicotiana benthamiana*, carrot calli, *Arabidopsis thaliana*, and wheat cv. Fielder (Lanes 2, 4, 7, and 11, respectively). In contrast, a specific target band of 2958 bp, consistent with the full-length NitNRCL1 sensor gene, was amplified from the corresponding *Agrobacterium*-mediated transiently infected or stably transformed materials (Lanes 3, 5, 8–10, and 12–14, respectively). These results confirmed the successful genomic integration and expression of the NitNRCL1 sensor gene in all tested plant species. Primers were designed to target the coding region of NitNRCL1, and all experiments were independently replicated three times with consistent and reliable outcomes.

https://doi.org/10.3390/xxxxx

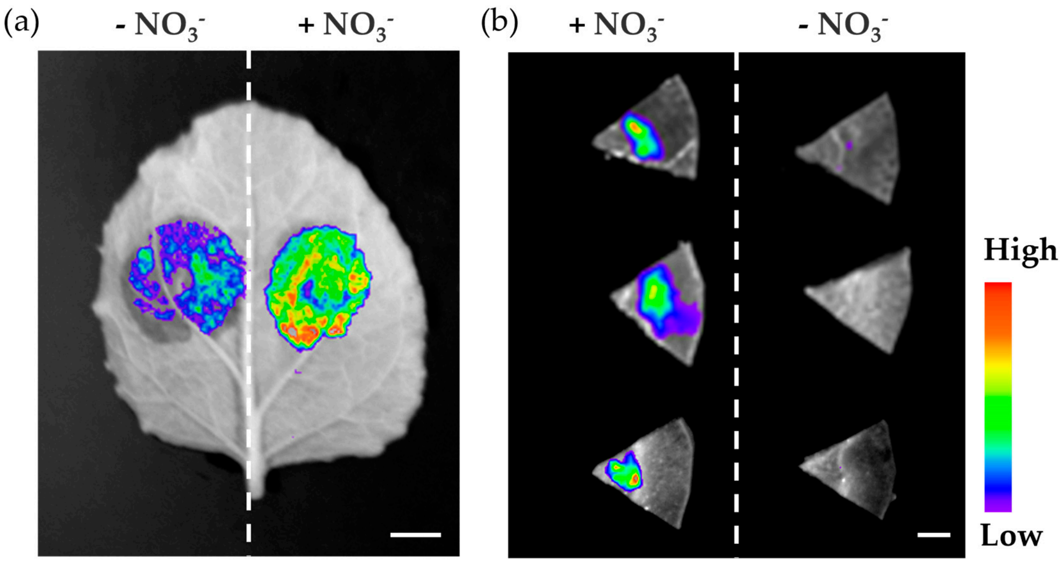

Figure S5. Functional validation of the NitNRCL1 sensor in diverse plant materials. Plant tissues harboring the NitNRCL1 sensor were generated via *Agrobacterium tumefaciens* GV3101 (pSoup-p19)-mediated transient transformation. For *N. benthamiana* leaves (a), the left side was treated with 5 mM KNO_3 and the right side with 5 mM KCl as a negative control. For carrot callus discs. (b), three calli on the left were incubated with 5 mM KNO_3 for 10 min, and three calli on the right with 5 mM KCl for 10 min. All samples were uniformly coated with 150 μg mL^{-1} potassium D-luciferin and imaged using a colorimetric imaging system. Consistent luminescent signals were detected in KNO_3-treated regions across all tested plant materials, demonstrating that the NitNRCL1 sensor confers sensitive and specific responsiveness to nitrate (NO_3^-) in diverse plant species. Scale bar, 1 cm.

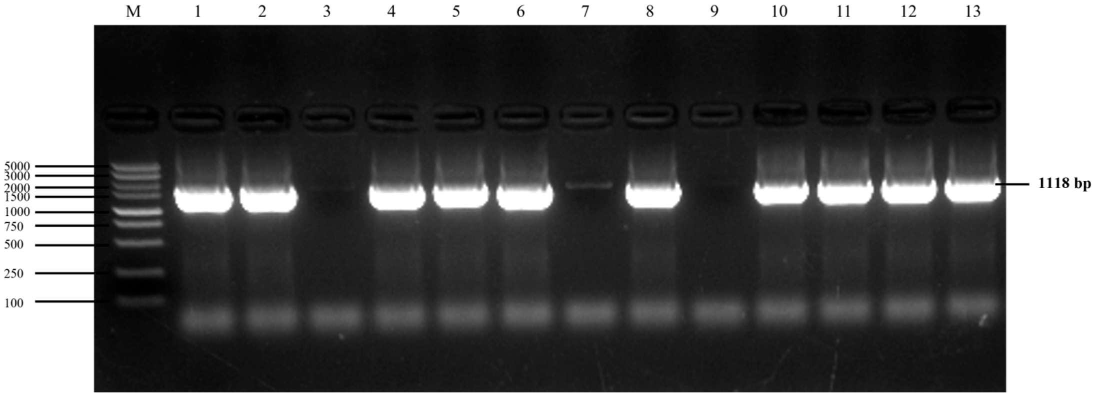

Figure S6. PCR identification of positive transgenic *Arabidopsis* thaliana plants. M: DNA Marker. Lanes 1 – 15 represent PCR identification results for individual transgenic Arabidopsis thaliana lines. The expected target amplicon size was 1118 bp. Twelve positive transgenic lines were successfully identified by PCR screening (see Figure 11), thereby providing essential core materials for subsequent functional validation of the NitNRCL1 sensor.

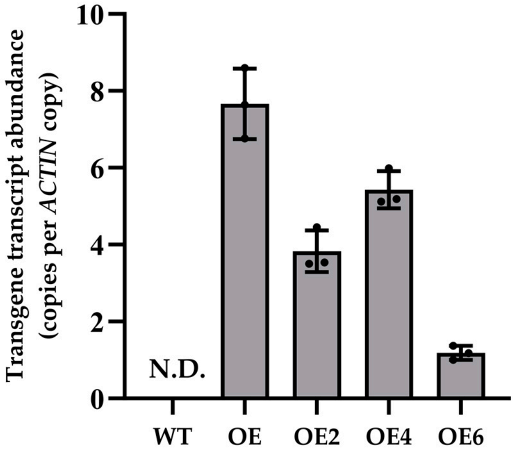

Figure S7. Absolute quantification of transcripts encoding the NitNRCL1 sensor in transgenic *Arabidopsis thaliana* overexpression lines. Absolute quantitative RT-PCR analysis was performed to determine the abundance of transcripts encoding the NitNRCL1 sensor in four representative independent transgenic lines (designated OE1, OE2, OE4, and OE6) and the wild-type (WT) control. A standard curve was generated using linearized plasmid DNA containing the target gene sequence, yielding an amplification efficiency of 98.56% and an R^2 value of 0.999. Expression levels are presented as the copy number of the exogenous gene per single copy of the *ACTIN* reference gene. The *ACTIN* standard curve exhibited an amplification efficiency of 103.4% (R^2 = 0.9997). No corresponding signal was detected in WT samples (signal below the lower limit of reliable quantification, indicated as N.D. in the figure). Data are shown as mean ± standard deviation (n = 3 independent biological replicates).

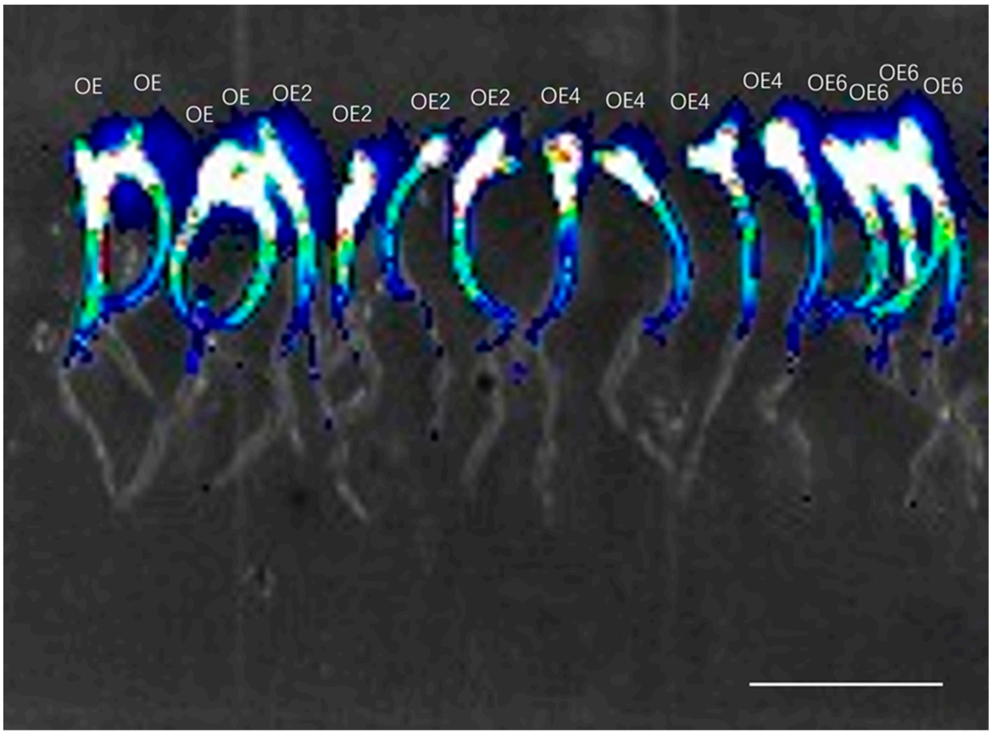

Figure S8. Response of transgenic *Arabidopsis* thaliana lines with varying expression levels to nitrate. Four transgenic Arabidopsis lines exhibiting distinct expression levels of the NitNRCL1 sensor were selected and treated with 10 mM NO_3^- by foliar spray. Luminescence intensity was monitored following nitrate application. All tested lines displayed robust luminescence responses to NO_3^-, indicating that functional nitrate sensing is retained across a range of transgene expression levels.

https://doi.org/10.3390/xxxxx

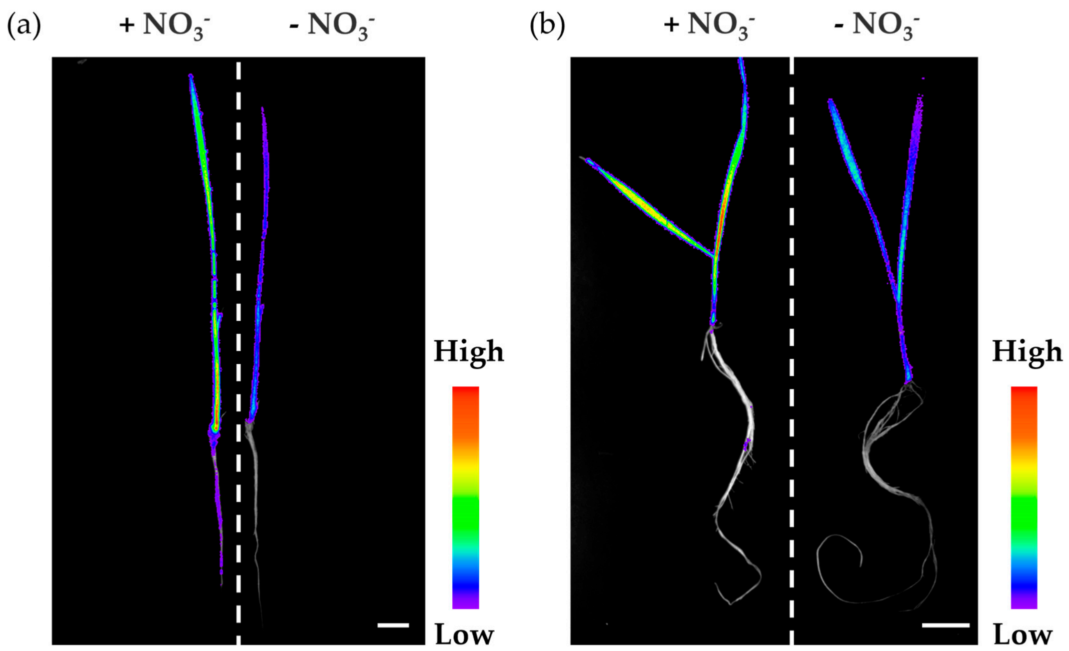

Figure S9. Functional validation of the NitNRCL1 sensor in rice and wheat. Transgenic rice cv. Zhonghua 11 (a) and transgenic wheat cv. Fielder (b) expressing the NitNRCL1 sensor were subjected to nitrate treatment: the left side of plant tissues was treated with 10 mM KNO_3 for 10 min, and the right side with 10 mM KCl for 10 min as a negative control. After uniform coating with 150 µg mL^{-1} potassium D-luciferin, samples were imaged using a luminescence imaging system. Strong luminescent signals were exclusively observed in the KNO_3-treated regions of both transgenic rice and wheat, confirming that the NitNRCL1 sensor maintains robust and sensitive nitrate (NO_3^-) responsiveness in monocot cereal crops (rice and wheat). Scale bar, 1 cm.

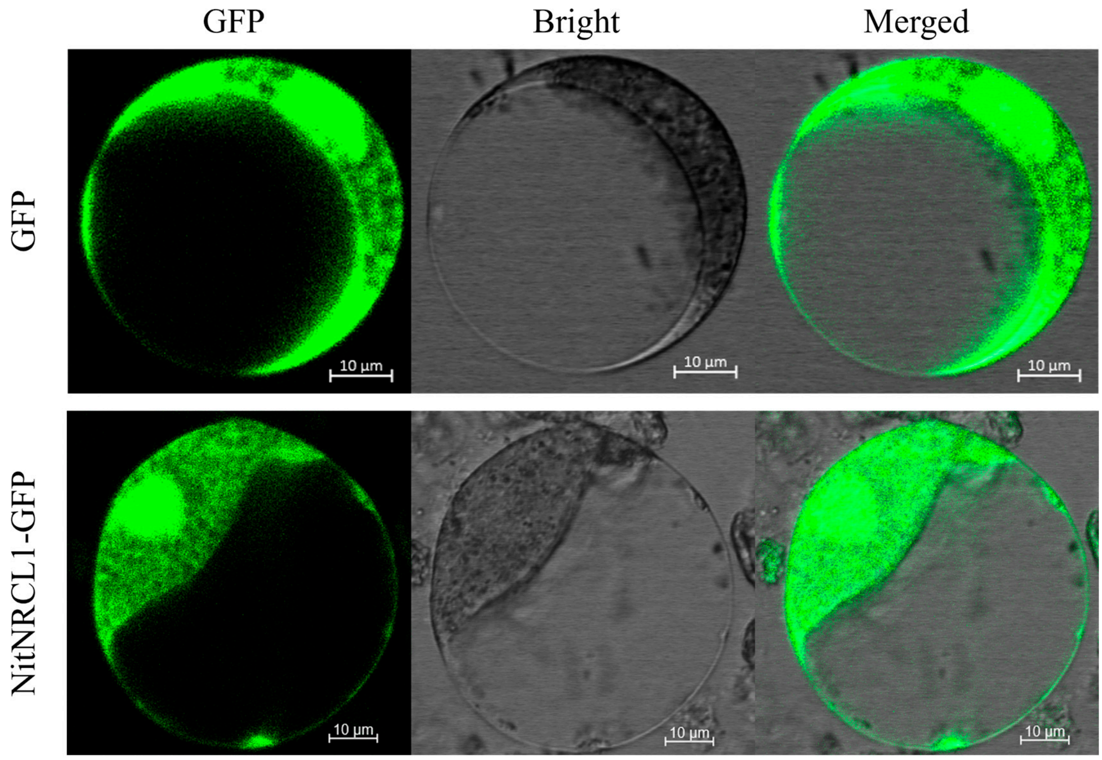

Figure S10. Subcellular Localization of NitNRCL1 in Wheat Protoplasts. Compared with the green fluorescent signal distribution of the 35S-GFP control, we observed that NitNRCL1 localized to the cytoplasm.

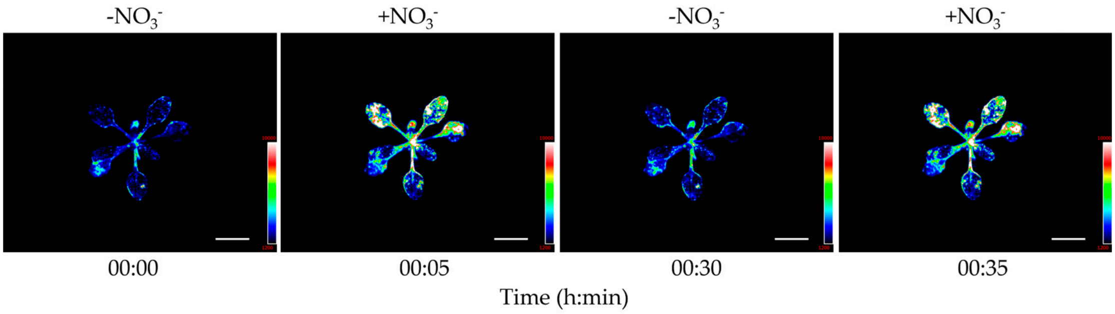

Figure S11. Reversibility of nitrate detection in transgenic *Arabidopsis* thaliana plants expressing NitNRCL1. Transgenic Arabidopsis plants grown under low-nitrogen conditions were treated with 10 mM KNO_3 by foliar spray. A robust luminescence signal was observed within 5 minutes of nitrate application. Immediately thereafter, KNO_3 was washed off, and the luminescence signal diminished substantially after 30 minutes. Subsequent re-application of 10 mM KNO_3 resulted in renewed luminescence emission. These observations confirm the reversible responsiveness of the NitNRCL1 sensor to nitrate availability in living plant tissues.

Table S1. List of primers used in this study.

Primer Name	Sequence (5′→3′)
pBWA-luc-F-GJ	CTTCTGTTGCAACTCCCCCGGGAGAGAACACGGGG-GACTTTGCAAC
pBWA-luc-R-GJ	GAGCTAGTTACACGGACTAGTAGAGTTACATCATCCATC
pCold-Ncluc-R	CGACAAGCTTGAATTCGGATCCAGGTAGAGGTCCAG-GAGCCGGACTAGTCCGGGCCTTTATGAGGATCTCTCTG
pCold-Cnluc-F	CCTGGATCCGAATTCGGCGGAGGTGGCAGCAAGCTTGAA-GACGCCAAAAACATAAAG
pCold-Ccluc-R	GCAGAGATTACCTATCTAGAGTTACATCATCCATCCTTGTC
pCold-nrtA-R-R	CTGCCACCTCCGCCGAATTCCGCCTTGATCGACTTGATC
pCold-nrtA-G-R	TGGCGTCTTCAAGCTTGGGTTCTGGCTTAG-GAGCGAATTCCGCCTTGATCGACTTGATC
pCold-nrtA-W-R	TGGCGTCTTCAAGCTTCGCCTTGATCGACTTGATC
pCold-nrA-F	CTGGACCTCTACCTGGATCCGGAACAGGCAGCTCCACCG
pCold-nrA-R	CTGCCACCTCCGCCGAATTCCCACTGGTCATGCGACTTG
pBWA(V)HS-F1	AGAGAACACGGGGGACTTTGCAAC
pBWA(V)HS-R1	GTACTGAAGACAGAGCTAGTTACA
TnrtA-qP-F	AGAGAACACGGGGGACTTTGC
TnrtA-qP-F	GCTTCTATGCCAAGTACGGC
pCold-F-0	CATATCGCCGAAAGGCACAC
pCold-R-0	GGCAGGGATCTTAGATTCTG
OsACTIN1-F	TCATGTCCCTCACAATTTCC
OsACTIN1-R	GACTCTGGTGATGGTGTCAGC
bar(123)F	CGGCGACGAGCCAGGGATA
bar(123)R	GCACCATCGTCAACCACTACAT
TaACTIN-F	CTATGTTCCCGGGTATTGCT
TaACTIN-R	AAGGGAGGCAAGAATCGAC

Table S2. Comparison of NitNRCL1 with existing nitrate biosensors.

Sensor Name	Detection Principle	Affinity (K_d)	Dynamic Range	Imaging Scale	Temporal Resolution	Application Scenario
NitraMeter3.0	FRET	~90 μM	Specific numerical range not provided; sensitivity reported in the micromolar to millimolar range, with in vitro maximum reached above 1mM nitrate.	Subcellular (nucleus/cytosol) and tissue level (Arabidopsis root apical meristem, transition zone, epidermis, cortex, endodermis, pericycle, etc.)	Seconds to minutes.	Real-time monitoring of nitrate dynamics in living roots of transgenic Arabidopsis.
mCitrine-NLP7	Split mCitrine	52±20 μM	100 μM to 10mM	Single-cell resolution	Rapid response. Reconstituted mCitrine fluorescence signal observed within 5 minutes after exogenous 10mM nitrate induction.	Real-time monitoring of nitrate dynamics in living roots of transgenic Arabidopsis.
FLIP-NT	FRET	5 μM	0.5-40 μM	Subcellular compartments	Seconds-level continuous monitoring.	Observation of nitrate dynamics in subcellular compartments in prokaryotic and eukaryotic systems.
NiTrac-NPF1.3	FRET	Not disclosed in the original text	Not disclosed in the original text	Single-cell (Yeast)	Seconds to minutes.	Validation of NPF1.3 function in single yeast cells and Xenopus laevis oocytes.
NiTrac1	Donor Quenching	High-affinity phase: 75.1±21 μM; Low-affinity phase: 3.8±2.6mM	Biphasic kinetics, covering both high-affinity and low-affinity ranges	Single-cell (Yeast, Xenopus oocytes)	Seconds-level.	Monitoring transporter activity in yeast.
NitNRCL1 (This study)	Split Luciferase (Bioluminescence)	12.9 μM	In vitro detection linear response 0.01 μM-50 μM; maximum reached at 5mM	Whole-plant macroscopic	Minutes-level.	Whole-plant, long-term nitrate dynamic tracing.

https://doi.org/10.3390/xxxxx

Appendix: Standardization Strategy for Luminescence Signal Detection

1. Substrate Concentration Control: A uniformly prepared working solution of luciferin potassium salt (1 mM) was used for all experiments.
2. Fixed Acquisition Parameters: The imaging system was cooled to -20°C, the exposure time was fixed at 3 min, and binning was set to 2×2 to ensure cross-experimental comparability.
3. Background Subtraction Method: An equal-area region devoid of plant material was selected as the background ROI. The target signal was calculated as: Raw Gray Value – Background Gray Value.
4. Pseudocolor Scale Standardization: A uniform signal intensity-to-pseudocolor mapping range was applied to all images within the same experiment.
5. Relative Quantification Strategy: Given that absolute luminescence intensity is influenced by factors such as substrate penetration efficiency and tissue thickness, relative changes (ΔRLU or fold change relative to control) were primarily used for quantitative comparisons.

https://doi.org/10.3390/xxxxx
